# Gene Polymorphism and Recurrent Atrial Fibrillation after Catheter Ablation: A Comprehensive Review

**DOI:** 10.31083/j.rcm2404119

**Published:** 2023-04-18

**Authors:** Meng-Fei Wang, Cong Xue, Shun-Yi Shi, Ling Yang, Zhen-Yan Zhu, Jian-Jun Li

**Affiliations:** ^1^Department of Cardiology, The Third Affiliated Hospital of Soochow University, The First People's Hospital of Changzhou, 213000 Changzhou, Jiangsu, China; ^2^State Key Laboratory of Cardiovascular Diseases, Fu Wai Hospital, National Center for Cardiovascular Diseases, Chinese Academy of Medical Sciences and Peking Union Medical College, 100037 Beijing, China

**Keywords:** gene polymorphism, atrial fibrillation, recurrence, catheter ablation

## Abstract

Atrial fibrillation (AF) is one of the most common cardiac arrhythmias, but its pathogenesis is still poorly understood. Catheter ablation is one of the most effective treatments for AF, but recurrence after ablation remains a challenge. There has been much research into the association of AF recurrence with several factors, including genetics. Over the past decade or so, significant advances have been made in the genetic architecture of atrial fibrillation. Genome-wide association studies (GWAS) have identified over 100 loci for genetic variants associated with atrial fibrillation. However, there is relatively little information on the systematic assessment of the genes related to AF recurrence after ablation. In this review article, we highlight the value of genetic polymorphisms in atrial fibrillation recurrence after catheter ablation and their potential mechanisms in the recurrence process to enhance our understanding of atrial fibrillation recurrence and contribute to individualized treatment strategies for patients with AF.

## 1. Introduction

Atrial fibrillation (AF) is one of the most common cardiac arrhythmias. The prevalence of AF varies slightly according to studies in different populations. Evidence suggests that the majority of AF in the general population is in the range of 1–5% [1, 2, 3]. According to the Framingham Heart Study (FHS), the prevalence of AF has increased threefold in the last 50 years [4]. The Global Burden of Disease project estimated the global prevalence of AF to be approximately 46.3 million in 2016 [5]. Patients with AF are at risk for cognitive decline, ischemic stroke, heart failure, myocardial infarction, and even death due to asynchronous atrial contractions, altered hemodynamics, and thromboembolism [6]. This has a tremendous negative impact on the patient, the whole family, and society.

**Fig. 1.**
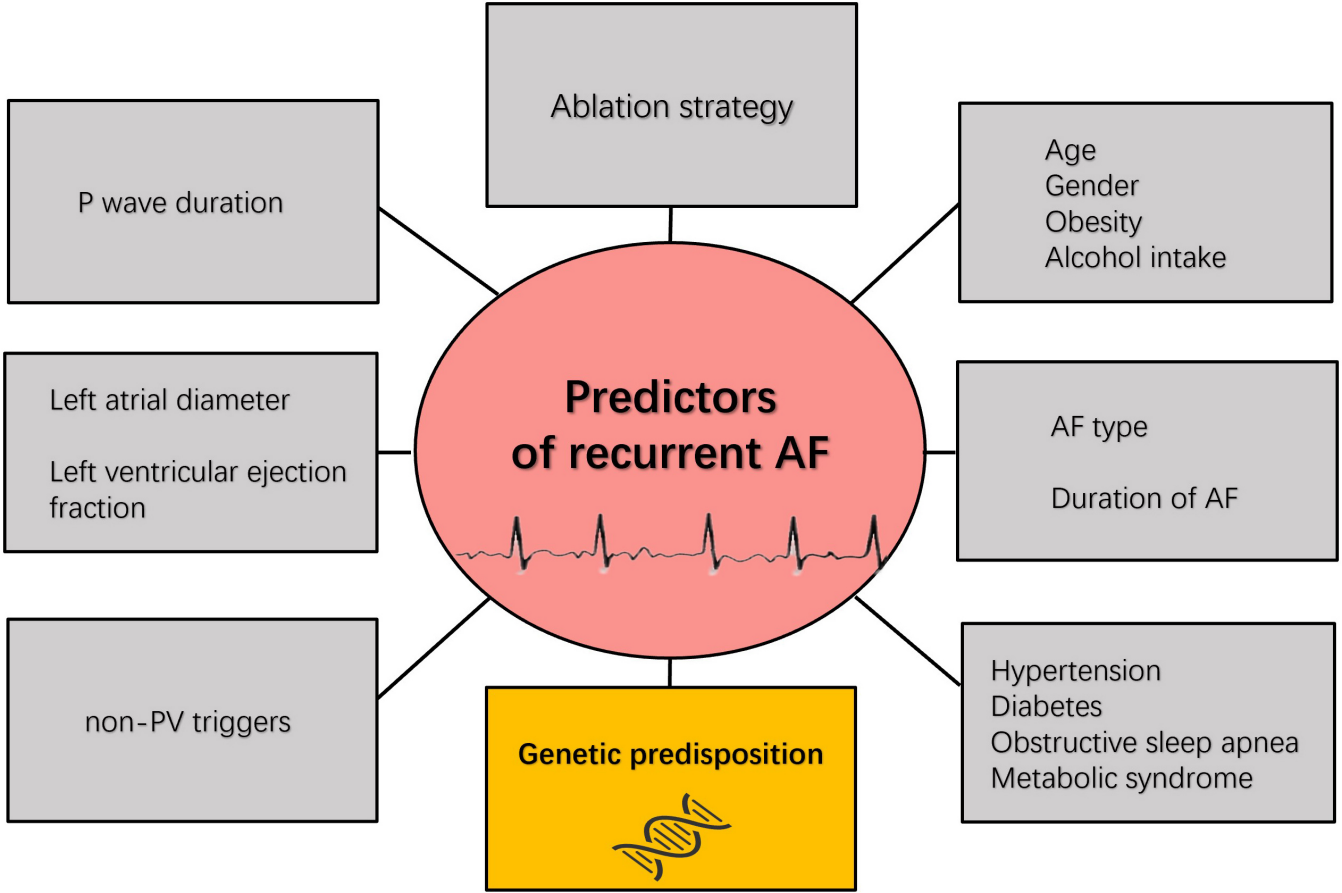
**Risk factors associated with recurrence of atrial fibrillation**. AF, atrial fibrillation; PV, pulmonary vein.

Catheter ablation is currently one of the main tools for treating atrial fibrillation. It is mainly used to improve symptoms and control the heart rhythm in patients who have failed antiarrhythmic drug therapy or are intolerant to drugs [7]. In the 2020 ESC AF guidelines, catheter ablation has been identified as the first-line treatment for rhythm control in AF, especially in patients with paroxysmal AF and those need to improve their symptoms [8]. However, arrhythmia recurrence, defined as AF/atrial tachycardia/atrial flutter for 30 seconds or longer three months after ablation, remains a significant limitation of catheter ablation, according to the 2017 consensus. Late recurrence occurs in 50% or more of patients within five years [9]. The common risk factors known to influence ablation success include sex, age, duration of AF, body mass index, left atrial diameter, degree of left atrial scarring, and coexisting conditions, including hypertension, metabolic syndrome, heart failure, and sleep apnea [10], as shown in Fig. 1.

**Fig. 2.**
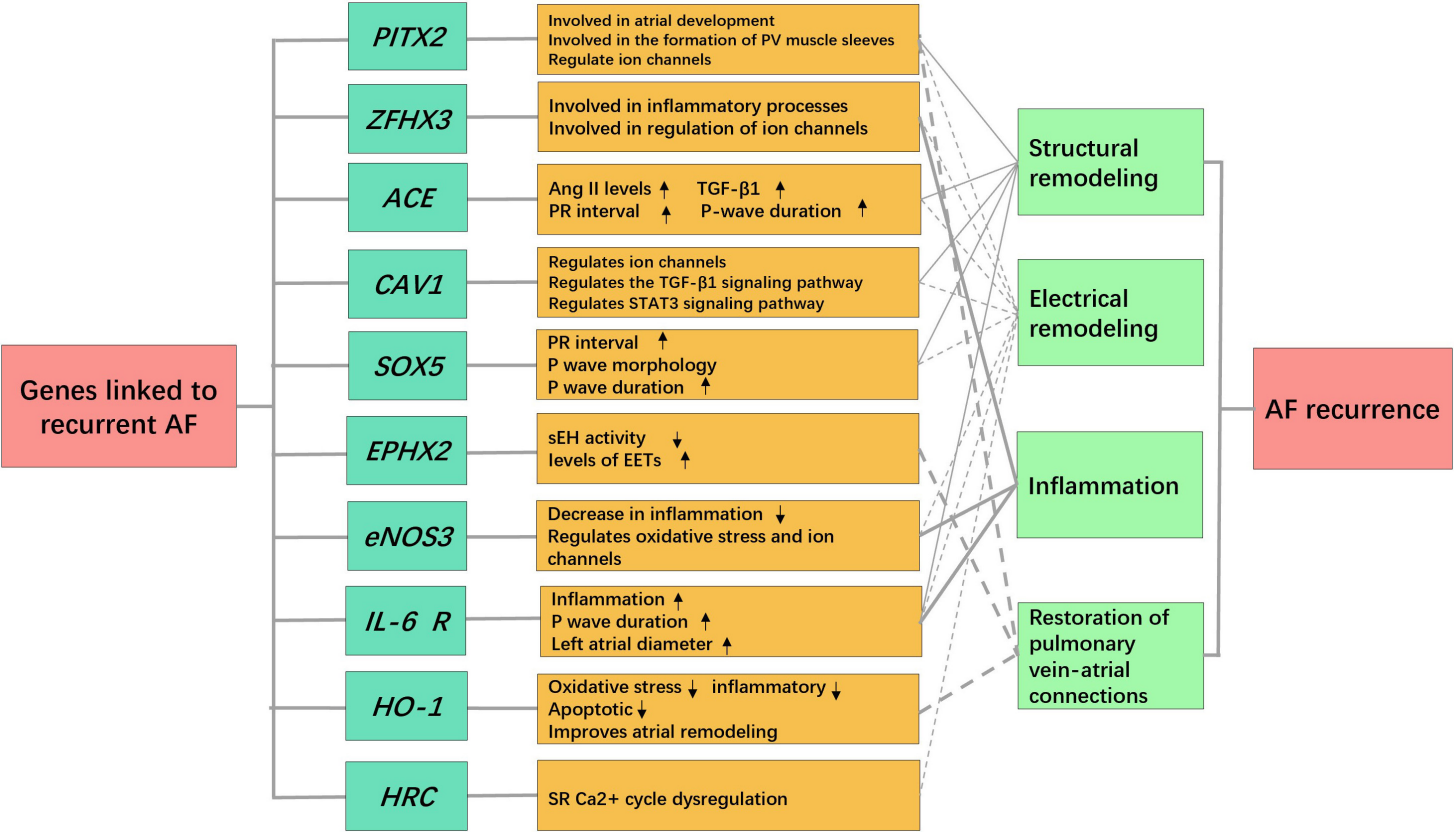
**Genes and potential mechanisms associated with recurrence of atrial fibrillation**. AF, atrial fibrillation; PV, pulmonary vein; *ACE*, angiotensin-converting enzyme; Ang II, angiotensin II; TGF-β1, transforming growth factor β1; *CAV1*, caveolin-1; STAT3, signal transducer and activator of transcription 3; sEH, soluble epoxide hydrolase; EETs, epoxyeicosatrienoic acids; *eNOS3*, endothelial nitric oxide synthase3; *IL-6 R*, interleukin-6 receptor; *HO-1*, heme oxygenase-1; SR, sarcoplasmic reticulum.

Genomics is a recently emerging field, and some evidence of family aggregation suggests that AF may be a genetically related disease [11]. This was further supported by a cohort study based on more than 5000 Icelandic patients with AF [12]. Over the past decades, more than 100 genes that are associated with AF have been identified. Some of these have been identified through classical linkage and family studies, while most were identified through functional or genome-wide association studies (GWAS). GWAS has identified many genetic variants (single nucleotide polymorphisms [SNPs]) associated with AF, and the role of genetic factors in the mechanism of AF is being increasingly recognized. Even a growing number of studies have found recurrence after AF ablation can be predicted using SNPs [13, 14, 15, 16, 17, 18, 19, 20, 21, 22, 23, 24, 25, 26]. Predicting the outcome of catheter ablation by individual genetic characteristics has excellent potential to guide AF treatment strategies. In the following, we review the genetic variants associated with recurrence after AF ablation (See Table 1 (Ref. [13, 14, 15, 16, 17, 18, 19, 20, 21, 22, 23, 24, 25, 26, 27, 28]) for relevant experimental studies and Fig. 2 for related genes and potential mechanisms).

**Table 1.** **Genetic polymorphisms associated with recurrence of atrial fibrillation**.

Author, year, and country	AF type	Study design	SNP	Adjacent gene	OR/HR	*p*-value	Key fingdings
					95% CI		
Husser, 2010, Germany [13]	drug-refractory paroxysmal or persistent AF	Cohort study	rs2200733	*PITX2*	ERAF: 2.108 (1.135–3.917)	0.018	The rs2200733 variant was independently associated with atrial fibrillation recurrence.
					LRAF: 2.458 (1.061–5.693)	0.036	
Chen, 2016, China [14]	lone AF; paroxysmal AF; persistent AF	Cohort study	rs2200733	*PITX2*	OR: 1.714	0.01	The rs2200733 variant was independently associated with atrial fibrillation recurrence.
					1.137–2.585		
Zhao, 2017, China [15]	paroxysmal or persistent AF	Cohort study	rs2200733	*PITX2*	HR: 1.766 (1.062–2.936)	0.028	The rs2200733 variant was independently associated with atrial fibrillation recurrence.
Choi, 2015, Korea [27]	paroxysmal or persistent AF	Observational study	rs2200733	*PITX2*	HR: 1.01 (0.80–1.26)	0.963	The rs2200733 variant was not associated with atrial fibrillation recurrence.
Hu, 2016, China [28]	paroxysmal or persistent AF	Observational study	rs2200733	*PITX2*	HR: 1.23 (0.87–1.76)	0.25	The rs2200733 variant was not associated with atrial fibrillation recurrence.
Park, 2017, Korea [16]	long-standing persistent AF (L-PeAF)	Observational study	rs2106261	*ZFHX3*	OR: 2.70 (1.41–5.14)	0.003	The rs2106216 variant was independently associated with good responders after radiofrequency ablation for L-PeAF.
Tomomori, 2018, Japan [17]	paroxysmal AF	Retrospective single-center study	rs2106261	*ZFHX3*	HR: 0.53 (0.29–0.98)	0.04	Patients with the minor (*T*) allele have a lower recurrence rate after ablation for paroxysmal atrial fibrillation.
Choi, 2015, Korea [27]	paroxysmal or persistent AF	Observational study	rs2106261	*ZFHX3*	HR: 0.86 (0.71–1.04)	0.128	The rs2106261 variant was not associated with atrial fibrillation recurrence.
Ueberham, 2013, Germany [18]	paroxysmal or persistent AF	Cohort study	I/D	*ACE*	OR: 2.25 (1.056–4.798)	0.036	*ACE DD* gene polymorphism was independently associated with atrial fibrillation recurrence.
Zhang, 2012, China [19]	lone AF	Prospective study	I/D	*ACE*	RR: 2.35 (1.10–5.04)	0.028	The *ACE* gene* DD* genotype had a 2.35-fold increased risk for AF recurrence compared with the *ACE* gene *II* + *ID* genotype.
Hong, 2020, Korea [20]	paroxysmal or persistent AF	Prospective study	rs3807989	*CAV1*	HR: 1.15 (1.02–1.31)	0.024	The rs3807989 variant was independently associated with atrial fibrillation recurrence.
Park, 2020, Korea [21]	early-onset AF, <40 y	Retrospective study	rs11047543	*SOX5*	HR: 2.723 (1.358–5.461)	0.005	The rs11047543 variant was independently associated with atrial fibrillation recurrence.
Wutzler, 2013, Germany [22]	drug-refractory AF	Cohort study	rs751141	*EPHX2*	12M: OR: 3.2 (1.237–8.276)	12M: 0.016	The rs751141 variant was independently associated with atrial fibrillation recurrence.
					24M: OR: 6.076 (2.244–16.451)	24M: <0.0001	
Shim, 2015, Korea [23]	paroxysmal or persistent AF	Cohort study	rs1799983 (Glu298Asp)	*eNOS3*	OR: 1.75 (1.07–2.86)	0.026	The rs1799983 variant was associated with early recurrence of AF (<3 months).
Wu, 2014, China [24]	drug-refractory AF or permanent AF	Retrospective study	rs4845625	*IL-6R*	ERAF: OR: 1.71 (1.20–2.38)	2.96 × ${\mathrm{10}}^{-3}$	The rs4845625 variant was independently associated with atrial fibrillation recurrence.
					LRAF: OR: 1.80 (1.28–2.55)	0.007	
Hu, 2013, China [25]	drug-refractory AF	Unclear	*HO-1* promoter GT repeats	*HO-1*	OR: 0.94 (0.90–0.99)	0.01	GT repeats polymorphism was independently associated with atrial fibrillation recurrence.
Amioka, 2019, Japan [26]	paroxysmal AF	Retrospective study	rs3745297 (T > G, Ser96Ala)	*HRC*	HR: 2.66 (1.32–5.0)	0.007	Ser96Ala was independently associated with atrial fibrillation recurrence.

AF, atrial fibrillation; ACE, angiotensin-converting enzyme; *CAV1*, caveolin-1; *eNOS3*, endothelial nitric oxide synthase 3; *IL-6 R*, interleukin-6 receptor; *HO-1*, heme oxygenase-1; SNP, single nucleotide polymorphism; OR, odds ratio; HR, hazard ratio; RR, relative risk; ERAF, early recurrence of atrial fibrillation; LRAF, late recurrence of atrial fibrillation. L-PeAF, long-standing persistent AF.

## 2. Gene Polymorphisms

### 2.1 PITX2 SNP rs2200733

rs2200733 is located in the intron of chromosome 4q25 region. In recent years, several studies have suggested that SNP rs2200733 (genotypes* CC*, *CT*, and *TT*) may be associated with the risk of recurrence after radiofrequency ablation of atrial fibrillation [13, 14, 15, 29, 30, 31, 32, 33]. One hundred ninety-five patients with AF were studied in a German population for the first time by Husser *et al*. [13]. SNP rs2200733 (4q25) and SNP rs10033464 (4q25) were found to be associated with early recurrence (within seven days after ablation) and late recurrence (three-six months after ablation) after radiofrequency ablation of AF. Patients with the associated allelic variant had a 2-fold and 4-fold risk of early and late recurrence, respectively, compared with patients without the variant. This finding indicates the potential role of genotyping in the prediction of atrial fibrillation ablation therapy and in the management of the peri-interventional period. Shoemaker *et al*. [29] studied 378 patients in the Vanderbilt AF registry for atrial fibrillation ablation. By multivariate analysis, patients containing the risk allele (*T*) at the rs2200733 locus had a 24% reduction in recurrence-free time survival compared with patients with normal genes (survival time ratio 0.76; 95% CI 0.6–0.95; *p* = 0.016). The recurrence rate after radiofrequency (RF) ablation in this group of AF patients was 87.5%, which was significantly higher than that in patients with the normal genotype (40%). This further suggests that SNP rs220073 has some predictive value in the development of atrial fibrillation or atrial tachycardia after radiofrequency ablation of AF. Chen *et al*. [14] conducted a detailed study of 235 Chinese Han patients with atrial fibrillation using COX regression analysis. They found that the rs2200733 *T* allele was associated with the risk of recurrence after radiofrequency ablation in patients with atrial fibrillation. The association remained significant after correcting for various factors such as age, sex, and hypertension. They also found that rs2200733 was positively correlated with the right atrial diameter (RAD) and right superior pulmonary vein (RSPV) diameter. Patients with the *TT* genotype had larger right atrial volumes and thicker right upper pulmonary vein internal diameters than patients with the *CC* genotype. Zhao *et al*. [15] arrived at a similar conclusion. They also found that the *TT* gene group in rs2200733 was more likely to have AF recurrence than the *TC* +* CC* group and that this gene group increased the risk of AF recurrence 1.766-fold; in multivariate regression analysis, rs2200733 was an independent risk factor for AF recurrence. Shoemaker *et al*. [30] showed that SNP rs2200733 was associated with recurrence after RF ablation in patients with AF in a dominant model multivariate analysis (HR 1.3, 95% CI 1.1–1.6, *p* = 0.011), while SNP rs10033464 (4q25) was not significantly associated with recurrence after AF ablation in a meta-analysis of patients with AF undergoing RF ablation at three medical centers in Germany. Similarly, Rattanawong *et al*. [31] included 3322 patients with AF, performed a meta-analysis of their data, and found a strong association between SNP rs2200733 and recurrence after AF ablation ( relative risk (RR) 1.45, 95% CI 1.15–1.83, *p* = 0.002). The meta-analysis by Hu and He also supported these results [32, 33].

Additionally, the rs2200733 polymorphism has been shown to have no significant effect on recurrence after RF ablation of AF. Choi *et al*. [27] found that SNP rs2200733 did not correlate with recurrence after atrial fibrillation ablation in 1068 patients with atrial fibrillation undergoing radiofrequency ablation in a Korean population; SNP rs6843082 (4q25), SNP rs2106261 (16q22), and SNP rs13376333 (1q21) were also confirmed to be uncorrelated with recurrence in patients with AF. However, Hu *et al*. [28] used 189 Chinese patients with AF as their study population. They found that the rs2200733 variant was not associated with AF recurrence, but combining the rs7193343 risk allele enhanced the predictive value of AF recurrence.

In summary, there is no agreement about the rs2200733 polymorphism affecting the outcome after AF ablation. How these variants exert functional effects and how they predict rhythm outcomes after catheter ablation are not clear. rs2200733 is located in the intron region of chromosome 4q25 [34], and the gene closest to it upstream, *PITX2*, plays a crucial role in cardiac development. In humans, *PITX2* is expressed in several isoforms, namely, *PITX2a*, *PITX2b*, *PITX2c*, and *PITX2d*. Only *PITX2c* is expressed asymmetrically in the heart and embryonic development. Deletion of this isoform may lead to malformations of the heart, abnormalities of the conduction system, and defects in cardiopulmonary muscle [35]. In earlier studies, it was found that *PITX2c* confers the morphology of the left atrium, and the deletion of *PITX2c* can give the left atrium the morphology of the right atrium [36]. Mouse embryos defective in the *PITX2* gene have ectopic sinus nodes. Hill *et al*. [37] further revealed changes in the composition of *PITX2*-deficient atrial cardiomyocytes and the basis for asymmetric left-right defects in atrial cardiomyocytes. The pulmonary vein sleeve is a myofibrillar connection between the pulmonary veins and the left atrium and plays an essential role in atrial fibrillation. *PITX2c* is involved in the formation, differentiation, and proliferation of the pulmonary vein sleeve, and *PITX2c*-deficient mice lack the pulmonary vein sleeve [38]. Based on this finding, we hypothesized that carriers of the chromosome 4q25 variant might have a different pulmonary vein phenotype, which may affect the outcome of pulmonary vein catheter ablation. In addition, *PITX2* regulates potassium and calcium channels; altered action potentials and changes in resting membrane potential are observed in *PITX2* mutant models [39]. Recently, *PITX2c*-deficient mutants were found to lead to early monoidal and metabolic defects and electrical instability in a zebrafish study model, which led to the development of arrhythmias [40]. Taken together, we found that *PITX2* is involved in the embryonic development of the atria, the formation of the pulmonary vein sleeve, and the regulation of atrial electricity. All of these structural and electrical remodelings may be risk factors for the recurrence of atrial fibrillation. Therefore, we speculate that rs2200733 may be involved in atrial fibrillation recurrence by regulating the expression of *PITX2c*.

### 2.2 ZFHX3 SNP rs2106261

The* ZFHX3 *gene (16q22) is the second gene highly associated with atrial fibrillation (AF), and it is associated with inflammation, stromal deposition, fibrosis, and atrial structure. Its SNPs are thought to increase susceptibility to AF [41, 42]. Interestingly, some recent reports suggest that SNP rs2106261 has predictive value for a good response after atrial fibrillation ablation. Park *et al*. [16] used multivariate logistic regression to study 141 patients with persistent AF and found that SNP rs2106216 had an excellent predictive value for patients with long-standing persistent AF without recurrence after radiofrequency ablation. In contrast, SNP rs7193343 (16q22) did not have this value. Tomomori *et al*. [17] genotyped rs2106261 (*CC*, *CT*, *CT*) and retrospectively studied 362 patients with paroxysmal AF. They found a lower AF recurrence rate in patients with the minor (*T*) allele using multivariate regression analysis (HR 0.53, *p* = 0.04). In addition, patients with paroxysmal AF with the minor allele of SNP rs2106261 (*TT* + *TC*) had lower levels of C reactive protein (CRP), neutrophil/lymphocyte (N/L) ratios, and interleukin 6 (IL-6) expression than patients with the *CC* type.

Similarly, studies on the rs2106261 polymorphism have had different findings. Choi *et al*. [27] found no correlation between SNP rs2106261 and postoperative recurrence in patients with atrial fibrillation in a study of 1068 patients with atrial fibrillation undergoing radiofrequency ablation in a Korean population. A meta-analysis by Jiang *et al*. [43] reached the same conclusion.

Thus, the impact of the SNP rs2106261 on postablation AF is also controversial. rs2106261 is located within the intron of the *ZFHX3* gene, which encodes *ZFHX3* ((or AT motif binding factor 1, (*ATBF1*)), a transcription factor containing multiple homologous structures and zinc finger motifs [44]. Although the *ZFHX3*gene is the second most crucial gene associated with AF, there is also uncertainty about its predictive value after ablation, and its underlying mechanisms have not been fully elucidated. (*ZFHX3* was involved in inflammation, ${\mathrm{Ca}}^{2+}$ regulation, and electrical remodeling in many *in vitro* and *ex vivo* studies. *In vitro*, by rapidly pacing HL-1 cells, the expression of ZFHX3 can be reduced, thereby reducing its binding to PIAS3 (STAT3 inhibitor) and increasing the transcriptional activity of signal transducer and activator of transcription 3 (STAT3) [45]. While STATs are the main downstream substances of inflammatory signaling, they may mediate the inflammatory response and play an essential role in the process of atrial fibrillation [46]. Downregulation of (*ZFHX3* has been reported to increase sarcoplasmic reticulum (SR) ${\mathrm{Ca}}^{2+}$ content, ${\mathrm{Ca}}^{2+}$ leakage, and ${\mathrm{Ca}}^{2+}$ transients; shorten action potential duration (APD); increase Kir3.4 and IKAch expression; and increase STAT3 and phosphorylated STAT3 in HL-1 atrial myocytes [47]. All of these factors contribute to forming and maintaining AF [48, 49, 50]. Huang *et al*. [51] also found that *ZFHX3* and *PITX2c *can regulate each other, with *ZFHX3* positively regulating *PITX2c* and *PITX2c* positively regulating *ZFHX3*. In addition, both *ZFHX3* and *PITX2c* regulate the expression of the *NPPA*, *TBX5*, and *NKX2.5* genes. The *NAAP* gene encodes cardiac natriuretic peptide (ANP), which governs cardiac ion channels and the autonomic nervous system and plays a vital role in cardiac electrophysiological activity [52, 53]. Both *TBX5* (encoding Tbox5) and *NKX2.5* (encoding NK2 transcription-related factors) also play a key role in cardiac electrophysiology and the development of AF [51, 54]. Recent studies have also found that the ZFHX3 transcription factor regulates INa channels [55]. This suggests that ZFHX3 may be involved in the recurrence mechanism of AF through its involvement in the inflammatory response as well as its regulation of ion channels. In addition, Husser *et al*. [56] and others found that *ZFHX3* was associated with left atrial internal diameter, leading to the inference that ZFHX3 may be involved in AF recurrence through structural remodeling; however, there are few studies in this area. Hwang *et al*. [57] even performed a retrospective study of 14 single nucleotides of *ZFHX3*. They found the *ZFHX3* gene polymorphisms (rs13336412, rs2106259, rs61208973, rs1858801, and rs12927436) were associated with extrapulmonary venous triggers but not with recurrence of AF. It is evident that although *ZFHX3* may be involved in the mechanism of atrial fibrillation recurrence, how these mononucleotides affect it remains to be further investigated.

### 2.3 Angiotensin-Converting Enzyme (ACE) SNP ACE I/D

The renin-angiotensin-aldosterone system (RAAS) is an important system that regulates cardiovascular function. Angiotensin-converting enzyme (ACE) is the key enzyme of the RAAS. Its primary function is to convert inactive angiotensin I (Ang I) into active angiotensin II (Ang II), which constricts blood vessels, raises blood pressure, and promotes the secretion of aldosterone from the adrenal cortex, of which aldosterone plays a role in sodium retention, water retention, and potassium excretion. The *ACE* gene is located on the long arm of chromosome 17 (17q23) and encodes ACE, which exerts biochemical effects. Ueberham *et al*. [18] found that *ACE DD* genotype was a risk factor for the recurrence of atrial fibrillation using multivariate logistic regression analysis in a study of 238 patients with atrial fibrillation. The risk of postoperative recurrence was 2.251 times higher in patients with the *DD* genotype than in those with the *ID* + *II* genotype. It was suggested that atrial fibrosis due to variants in the *ACE* gene might be associated with recurrence after AF ablation. Zhang *et al*. [19] studied 193 patients with isolated AF in a Chinese Han population. They found a 2.35-fold increased risk of recurrence in patients with *ACE DD* genotype AF compared with patients with *ID* + *II* genotype using a multivariate logistic regression analysis. This study also found a larger left atrial internal diameter in patients with the *DD* genotype, suggesting that the effect of the *ACE* gene I/D polymorphism on isolated AF recurrence may be through left atrium diameter.

In past studies, a large body of evidence has shown that the RAAS is associated with cardiovascular disease. It has pro-inflammatory, antioxidant, and fibrotic effects. The mechanism of the *ACE* gene for recurrence after atrial fibrillation ablation is unclear. The *ACE* gene has a 287-bp repetitive sequence fragment in intron 16, and this fragment is often present as an insertion (I) or deletion (D) of this polymorphism [58]. Studies show elevated serum ACE levels and cardiac ACE activity in people with the *ACE DD* genotype [59]. Therefore, angiotensin II levels will also increase accordingly. Evidence suggests that angiotensin II induces fibroblast proliferation and collagen synthesis by increasing transforming growth factor β1 (TGF-β1) expression, leading to cardiac fibrosis [60]. Liu *et al*. [61] also showed that platelet-derived TGF-β1 promotes AngII-induced atrial fibrosis and the development of atrial fibrillation. They also found that structural remodeling and electrical conduction induced by AngII could be attenuated by platelet inhibitors [61]. Of course, increased TGF-β1 expression has been observed in animal models of atrial fibrillation [62]. Furthermore, a study by Watanabe *et al*. [63] found that the *ACE D* allele was associated with cardiac conduction abnormalities, as evidenced by prolonged PR intervals and P-wave duration. These two factors are also recognized as risk factors for increased recurrence of AF [64, 65]. Therefore, several lines of evidence suggest that patients with the *ACE DD* genotype may have more myocardial fibrosis and cardiac conduction abnormalities than patients with the *II*/*ID* genotype and are more likely to have AF recurrence.

### 2.4 *Caveolin-1 (CAV1)* SNP rs3807989

rs3807989 is located in an intron on the *Caveolin-1 (CAV1)* gene, which is associated with PR interval and atrial fibrillation in European populations [66, 67]. Chen *et al*. [68] first found similar results in a Chinese population, and they found that SNP rs3807989 in *CAV1* was related to lone AF. A prospective study of 1722 patients who underwent catheter ablation found that the rs3807989 polymorphism was associated with AF recurrence. In addition, they corrected for confounding factors such as age, sex, LA diameter, LA volume, and QTc interval in 1290 AF patients entered into a Mendelian randomization analysis model. The PR interval was found to be highly associated with recurrent AF in terms of *CAV1* (rs3807989, HR 1.04, 95% CI 1.01–1.07, *p* = 0.006) [20]. Ulus *et al*. [69] also found that the SNP rs3807989 predicted AF recurrence using multiple COX regression model analysis in Turkish patients with atrial fibrillation undergoing frozen balloon pulmonary vein isolation (rs3807989 G: OR 4.5, 95% CI 1.04–19.31, *p* = 0.043).

The caveolae are characterized by a 50–100 nm invagination of the cell surface plasma membrane. The fossa proteins are the major components of the fossa and are its main structural proteins. There are three isoforms of caveolin: caveolin-1, caveolin-2, and caveolin-3. Caveolin-1 is usually expressed in fibroblasts, endothelial cells, and smooth muscle cells [70]. The *CAV1* gene encodes caveolin-1. The mechanism of the *CAV1* gene in atrial fibrillation recurrence is also unclear. First, caveolin-1 affects ion channels and may have an important role in the development of AF. Caveolin-1 interacts with both the potassium channel Kir2.1 [71] and the pacemaker (HCN) channels HCN4 [72]. Second, caveolin-1 has also been found to have an important role in antifibrosis. Zhang *et al*. [73] demonstrated that caveolin-1 inhibits the TGF-β1 signaling pathway by regulating mitsugumin53 (MG53) and exerts an anti-fibrotic effect. Similarly, caveolin-1 depletes activation of the STAT3 signaling pathway and TGF-β1 signaling pathway, which leads to extracellular matrix deposition [74]. These results suggest caveolin-1 is involved in atrial fibrillation recurrence through its effects on ion channels and myocardial fibrosis. Therefore, we speculate that rs3807989 may be interested in atrial fibrillation recurrence through down-regulation of caveolin-1 expression, but the exact mechanism remains to be further investigated.

### 2.5 SOX5 SNP rs11047543

rs11047543 is located near the *SOX5* gene, which encodes a transcription factor that plays an important role in cellular regulation through its transcriptional activity [75]. Park *et al*. [21] retrospectively analyzed 89 patients (age <40 years) with early-onset AF with at least 12 months of follow-up and found by multivariate analysis that the SNP rs11047543 for *SOX5* (12p12) was an independent predictor of recurrence after AF ablation. Moreover, the recurrence rate was significantly higher in patients with the rs11047543 *GA* genotype than in patients with the *GG* genotype (72.2% vs 37.7%) in this study [21].

There are few reports on the association of rs11047543 with AF. Olesen *et al*. [76] found that SNP rs11047543 was associated with early-onset AF. Seifert *et al*. [77] found that SNP rs11047543 was associated with P-wave morphology. A meta-analysis based on 1010 patients showed that prolonged P-wave duration was significantly associated with postoperative recurrence of AF and that 149.5 ms was its potential threshold value [65]. More recently, a meta-analysis based on significant European and Asian populations similarly proposed a correlation between P-wave duration and AF and found associated genes suggesting a role of genetic factors in P-wave duration [78]. Although P-wave morphology and P-wave duration are not identical concepts, both are responsive to impulse conduction in the atria, and abnormalities in atrial conduction may be a factor in the recurrence of AF.

In addition, six single-nucleotide polymorphisms, including *SOX5* SNP rs11047543, were associated with the PR interval in a genome-wide association meta-analysis [79]. Park *et al*. [64] found that a prolonged PR interval predicted atrial fibrillation recurrence. Additionally, they found that as the PR interval increased, the P-wave duration lengthened, the left atrial volume increased, and the intra-left atrial voltage decreased, suggesting that the PR interval may be associated with late atrial remodeling [64]. Another study based on GWAS data found that* SOX5* was associated with PR interval, and *SOX5* was significantly associated with the left atrial internal diameter and left atrial low voltage region [80]. PR interval represents intra-atrial conduction and atrial to interventricular conduction time, and its prolongation can increase the risk of arrhythmia and is influenced by genetic factors [81]. Thus, we speculate that rs11047543 may promote AF recurrence by regulating *SOX5*, affecting P-wave morphology, P-wave duration, and the PR interval.

### 2.6 EPHX2 SNP rs751141

Epoxyeicosatrienoic acid (EET) is a product of cytochrome P450 cyclooxygenase and exerts cardioprotective effects in various experimental models [82]. EET has been shown to modulate inflammatory pathways (reducing inflammatory response) [83], cardiac ion channels [84, 85] (inhibiting Na and L-Ca ion channels), and oxidative stress [86], which all have effects on AF susceptibility. EET has also been shown to protect mitochondria, resist apoptosis, fight cardiac fibrosis, and combat cardiac hypertrophy [87, 88]. EET is metabolized by soluble epoxide hydrolase (sEH) to a less biologically active diol (dihydroxyeicosatrienoic acid, DHET) [89]. Most experimental models demonstrate that sEH inhibition also benefits the heart [90, 91, 92]. Several exonic variants on *EPHX2* (the gene encoding sEH) have been shown to increase or decrease sEH activity *in vivo*, including rs 751141 [93]. Wutzler *et al*. [22] recruited 218 patients with drug-refractory atrial fibrillation undergoing catheter ablation treatment. The *EPHX2* gene SNP rs751141 was found to be an independent predictor of AF recurrence after catheter ablation by multivariate analysis. The presence of its variants increased the risk of recurrence by 3-fold at 12 months and 6-fold at 24 months after catheter ablation, respectively [22].

From the above description, we know that sEH inhibition and increased EET activity benefit the heart. In cellular experiments, sEH activity was lower in cells containing the rs751141 mutant because its mutation may have affected the stability of she [93]. It is assumed that patients containing this mutant have higher levels of EETs in their blood and tissues. Therefore, the protective effect of rs751141 is what we expected, but the results are surprising. On the other hand, some experimental and clinical findings have conflicting results with reduced sEH activity, which has a protective effect on the heart. In the CARDIA study, the rs751141 allele was associated with an increased risk of coronary calcification in African Americans [94]. Another study found no effect of the rs751141 allele on either ischemic stroke or ischemic heart disease in a Danish population [95]. We think that the positive results of sEH inhibition and increased EET levels in experimental models (inflammation, ischemia, and heart failure) may not fully apply to arrhythmias. There is certainly no doubt about the protective role of EETs in cardiac injury. EET accelerates wound neovascularization and healing [96], and this property may prevent scar formation between the pulmonary veins and the atria such that the recurrence of AF is higher after ablation. On the other hand, the modulation of cardiac ion channels by regional heterodimers of EETs also impacts AF recurrence, but more studies are needed to confirm this [84, 85, 97, 98, 99]. Therefore, we conjecture that the rs751141 variant may affect AF recurrence by regulating sEH activity *in vivo*, affecting EET levels, although more studies are needed to prove the exact process.

### 2.7 eNOS3 SNP rs1799983 

The *eNOS* gene is located on chromosome 7 [100], contains 26 exons, and encodes a nitric oxide synthase (NOS) protein [101, 102]. There are three isoforms of nitric oxide synthase: NOS1, NOS2, and NOS3. NOS3 is also known as endothelial NOS (eNOS). In the heart, NOS1 and NOS3 are usually expressed [103]. On exon 7 of the *eNOS* gene, there is a clinically relevant variant called the G894T variant (*eNOS3* SNP rs1799983) that corresponds to the glutamate-aspartate substitution (Glu298Asp) [104]. The rs1799983 variant (Glu298Asp) is associated with reduced basal nitric oxide (NO) production [105]. Shim *et al*. [23] recruited 500 patients with atrial fibrillation for radiofrequency ablation therapy. At a mean follow-up of 17 months, SNP rs1799983 of the *eNOS3* gene was associated with early AF recurrence (within three months). After multiple logistic regression analyses, SNP rs1799983 was an independent predictor of early atrial fibrillation recurrence and was not associated with late recurrence [23].

Nitric oxide synthase (eNOS) and its product nitric oxide (NO) play an essential role in vasodilation and maintenance of cardiovascular homeostasis. NOS produces NO in the heart, affecting almost all mechanotransduction pathways in cardiac myocytes. It mediates mechanosensing, mechanical-electrical feedback (by modulating ion channel activity, including $K^{+}$, ${\mathrm{Na}}^{+}$, and ${\mathrm{Ca}}^{2+}$ channels, etc.), and ${\mathrm{Ca}}^{2+}$ handling [106, 107]. eNOS and NO contribute to atrial myocardial superoxide production [108]. In addition, eNOS can interact with CAV1, inhibiting eNOS activity and reducing NO release, as evidenced by enhanced endothelium-dependent diastolic function in *CAV1*-deficient mice. In contrast, eNOS can also act directly on CAV1; this is only a result of *in vitro* experiments and is not based *in vivo * [109].

The exact mechanism by which the *eNOS3* SNP rs1799983 is associated with early recurrence of AF is unclear, but the possibilities have been considered. First, acute inflammatory responses due to tissue damage formation should be widely present after catheter ablation of atrial fibrillation. In turn, these inflammatory responses cause the activation of signaling pathways that contribute to structural, electrical, and mechanical heterogeneity of atrial muscle, thereby promoting arrhythmogenesis [110]. The detrimental effects of inflammation and oxidative stress on atrial electrical and structural remodeling were mentioned in the summary of Karam *et al*. [111], which suggested that oxidative stress and inflammation could be targeted through some pathways to help control atrial fibrillation. The rs1799983 variant (Glu298Asp) is associated with reduced basal nitric oxide (NO) production [105]. In contrast, Shim *et al*. [23] found a higher rate of early recurrence in patients containing the rs1799983 variant; this may be due to reduce NO activity leading to slower inflammatory regression after atrial fibrillation ablation (RFCA), thus promoting recurrence of AF. Second, dysfunction of NOS and NO in atrial oxidative damage and electrical remodeling may also contribute to the reproduction of AF.

### 2.8 IL-6R SNP rs4845625 

There is much evidence that inflammation plays a vital role in the pathophysiology of AF, including C-reactive protein, interleukins, and tumor necrosis factor-α [112]. In a meta-analysis, a variant of intron rs4845625 of the interleukin-6 receptor (*IL-6 R*) gene was found to be associated with AF in Caucasians [113]. At the same time, Smit *et al*. [114] found baseline levels of IL-6 to be an independent predictor of early recurrence of AF by multifactorial analysis (HR 1.3, 95% CI 1.0–1.7, *p* = 0.02). Wu *et al*. [24] studied 278 patients with AF in a retrospective study in a Chinese Han population with approximately one year of follow-up. They found that the SNP rs4845625 for* IL-6R* was associated with the recurrence of AF both early (4 weeks post-ablation) and late (3–12 months post-ablation). This relationship remained significant after correction for hypertension, age, sex, and diabetes mellitus. And the probability of recurrence was higher in patients with the *T* allele than in patients with the *C* allele in the dominant, recessive, and additive models.

The mechanism of how the rs4845625 polymorphism is involved in recurrence after atrial fibrillation ablation is still not well understood. Inflammation is one of the mechanisms by which AF occurs. Frustaci *et al*. [115] performed biopsies on the hearts of 12 patients with lone AF and found inflammatory lymphomonuclear infiltration and peripheral cell necrosis in their atrial myocytes, which was not observed in patients with sinus rhythm. This was supported in an animal model with increased susceptibility to AF in a sterile pericardial model in dogs, where the incidence of AF was reduced by cortisol steroid hormone treatment and where the inflammatory markers (IL-6, CRP, and TNF-α) were significantly lower in this study group than in the control group [116]. Specific localization of C-reactive protein was also found in the cytoplasm of atrial myocytes in patients with AF [117]. Several studies based on European and Asian populations have demonstrated that elevated indicators of inflammation can increase the risk of atrial fibrillation [118, 119, 120]. All of the above evidence supports the possible involvement of inflammation in the development of AF.

There is certainly some evidence that inflammation is an essential mechanism for recurrence after atrial fibrillation ablation. A meta-analysis based on large-scale data found that high-sensitivity C-reactive protein and interleukin-6 were strongly associated with atrial fibrillation recurrence [121]. Elevated serum MMP-2 levels were an independent predictor of atrial fibrillation recurrence [122]. A study of marathon runners found increased P-wave duration after exercise accompanied by increased levels of IL-6, CRP, and neutrophils, suggesting that acute changes in inflammatory cytokines may be associated with interatrial cell conduction [123]. Another study suggested that inflammation is associated with atrial structural remodeling. They found that IL-6 and CRP were associated with increased left atrial internal diameter [124]. From this, IL-6 might increase the recurrence of AF through electrical and structural remodeling. Nevertheless, unfortunately, Wu *et al*. [24] did not find a relationship between variant rs4845625 and serum IL-6 levels. The reason was that they did not use serum specimens from AF patients, only serum samples from the healthy population, and the sample size was minimal. Therefore, using sera from the AF population may yield different results if replicated in a larger cohort. Thus the mechanism of SNP rs4845625 involvement in the risk of recurrence after AF ablation also needs further exploration.

### 2.9 Heme Oxygenase-1 GT Repeat Polymorphism 

Heme oxygenase-1 (HO-1) is a vital rate-limiting enzyme in the degradation of heme that degrades heme to iron ions, carbon monoxide (CO), and biliverdin; the latter is then converted to bilirubin by biliverdin reductase. HO-1 and its products can be activated by various oxidizing substances and act as an antioxidant system to protect the body [125]. Hu *et al*. [25] recruited 205 patients with drug-refractory AF undergoing radiofrequency ablation. They used multifactorial regression analysis and found that the *HO-1* promoter polymorphism (GT repeat sequence) was associated with recurrence after paroxysmal AF ablation, with shorter GT repeat sequences in the recurrence group than in the nonrecurrence group [25].

HO-1 is a protective factor with anti-inflammatory, antioxidant, and anti-apoptotic effects [126, 127]. In transgenic mice, HO-1 prevents oxidative stress in myocardial tissue and improves smooth intimal proliferation and inflammatory responses in coronary arteries [128]. In an infarct model, human HO-1 (h HO-1) was injected intracardially into the rat myocardium by adenovirus, causing an increase in HO-1 expression. They found a significant decrease in infarct size and myocardial lipid peroxidation levels, pro-apoptotic Bax and pro-inflammatory interleukin-1β proteins, and an increase in anti-apoptotic Bcl-2 protein levels [129]. Another recent study found that HO-1 has a protective effect on atrial structural remodeling. Hsu *et al*. [130] found that patients with shorter *HO-1* promoter GT repeat sequences had HO-1 overexpression, while patients with a reduced degree of atrial oxidative stress decreased collagen fiber production and reduced myogenic fiber degradation. Yeh *et al*. [131] found similar results in animal experiments.

Radiofrequency ablation causes a conduction block between the pulmonary veins and the atria through thermal injury. The GT repeat sequence was shorter, and the HO-1 expression level was higher in the recurrence group. HO-1 plays a vital role in antioxidative stress, anti-inflammation, anti-apoptosis, and improvement of atrial remodeling; thus, *HO-1* may help restore electrical conduction between the pulmonary vein and atrial myocytes and reduce scar formation between them by reducing the damage to cardiomyocytes by RF ablation, thus leading to AF recurrence. In addition, the experiment by Hu *et al*. [25] did not find an effect of *HO-1* promoter polymorphism on the outcome of persistent atrial fibrillation recurrence. Because they considered more factors affecting persistent AF recurrence, including more extensive atrial fibrosis, more atrial myocardial remodeling, and extrapulmonary vein trigger foci and had a limited number of cases, their ability to statistically analyze persistent AF cases was limited [25].

### 2.10 HRC SNP rs3745297 (T > G, Ser96Ala)

Sarcoplasmic reticulum (SR.) ${\mathrm{Ca}}^{2+}$ overload due to protein kinase A (PKA) hyperphosphorylation of the cardiac ryanodine receptor (RyR2) plays an essential role in the development and maintenance of atrial fibrillation [132]. *HRC* is a vital regulator of SR ${\mathrm{Ca}}^{2+}$ homeostasis in cardiac myocytes [133]. A study showed that the human *HRC* variant Ser96Ala was overexpressed in cardiomyocytes by gene transfer to adenovirus, resulting in increased SR ${\mathrm{Ca}}^{2+}$ overload and ${\mathrm{Ca}}^{2+}$ spark frequency [134]. Japanese scholar Amioka *et al*. [26] recruited 334 patients with paroxysmal AF who underwent radiofrequency ablation and found that *HRC* SNP rs3745297 (Ser96Ala) was an independent risk factor for AF recurrence by multifactorial regression analysis. The frequency of minor allele *G* was significantly higher in the recurrence group than in the nonrecurrence group (allele frequency model OR 1.8, *p* = 0.006; recessive model OR 3.55, *p* = 0.0009). Only 16 of the 57 recurrent patients in this study underwent secondary ablation. Their trigger sites were nine pulmonary veins (PV), one superior vena cava (SCV), one septum, and five unknown sites. Six of these patients had the *HRC* variant (Ser96Ala), but, unfortunately, no correlation was found between the *HRC* variant and the AF trigger site [26].

The *HRC* SNP rs3745297 (Ser96Ala) has been well studied in the field of heart failure and ventricular arrhythmias [134, 135]. Amioka was the first to identify the* HRC* SNP rs3745297 (Ser96Ala) associated with AF recurrence in this observational study. However, the mechanism of recurrence in patients with paroxysmal AF with the *HRC* Ser96Ala variant has not been clarified. The conjectured mechanisms are considered as follows. First, nonpulmonary vein trigger foci are a risk factor for AF recurrence. However, the frequency of *HRC* variants was similar in patients with AF of the pulmonary vein and nonpulmonary vein origin in the study by Amioka *et al*. [26], and the origin of AF recurrence was not found to be associated with *HRC* Ser96Ala. Therefore, the effect of *HRC* variants on nonpulmonary vein trigger foci, and thus the mechanism of AF recurrence may not be explained. Second, changes in ${\mathrm{Ca}}^{2+}$ and ${\mathrm{Ca}}^{2+}$ processing play an important role in the mechanism of triggering and maintaining AF [136]. Ion channels, including L-type ${\mathrm{Ca}}^{2+}$, and Ito, are involved in the alteration of the atrial nonresponse period that maintains AF and thus have an important role in the electrical remodeling of AF [137]. In addition, tachycardia-induced ${\mathrm{Ca}}^{2+}$ handling plays an essential role in the electrical remodeling of the atria [138]. Because the *HRC* variant Ser96Ala causes dysregulation of SR ${\mathrm{Ca}}^{2+}$ cycling [139], we speculate that patients with paroxysmal AF who possess the *HRC* Ser96Ala variant may be continuously exposed to ${\mathrm{Ca}}^{2+}$ overload, leading to inactivation of L-type ${\mathrm{Ca}}^{2+}$ channels and shortened action potentials, causing electrical remodeling of the atria and ultimately leading to AF recurrence.

## 3. Gene Polymorphisms and New-Onset Atrial Fibrillation

New-onset atrial fibrillation (new AF) is defined as atrial fibrillation that occurs during critical illness without a known previous history of atrial fibrillation. Myocardial ischemia, myocardial strain, electrolyte disturbances, sympathetic activation, and oxidative stress may be the causes of new AF [140, 141]. Genetic genes have been studied less frequently and with inconsistent results in this population of new AF. Kerchberger *et al*. [140] identified several single nucleotide polymorphisms associated with new AF in 1936 critically ill ICU patients, showing after controlling for clinical factors that rs3853445 (near *PITX2*, OR 0.47, 95% CI 0.30–0.73, *p* = 0.001) and rs12415501 (in *NEURL*, OR 1.72, 95% CI 1.27–2.59, *p* = 0.01) were associated with new-onset atrial fibrillation. Adding genetic factors to clinical factors in multivariate regression models helps identify new-onset atrial fibrillation [140]. Siebert *et al*. [142] conducted a prospective study of 203 patients with coronary artery disease who underwent coronary artery bypass grafting and did not find that the Scal polymorphism in the *ANP* gene predicted new-onset atrial fibrillation after coronary artery bypass. Kertai *et al*. [143] similarly selected coronary artery bypass graft patients and found that only SNP rs10504554, located in the *LY96* intron region, was associated with a reduced risk of new-onset postoperative atrial fibrillation based on a genome-wide association study (OR 0.48, 95% CI 0.34–0.68, *p* = 2.9 × ${\mathrm{10}}^{-5}$). Plante *et al*. [144] identified two single nucleotide polymorphisms (R87Q and P307S) in the voltage-gated channel hKv1.5 in a French-Canadian coronary artery bypass grafting population that altered the expression of gating processes and hKv1.5 channels; and patients with new-onset atrial fibrillation were more likely to have this polymorphism compared with controls (6.25% vs 3.37%; *p* = 0.42). A retrospective study by Kiliszek *et al*. [145] analyzing patients with an acute heart attack from two major centers in Poland found that SNP rs10757278 was associated with the development of new AF in such patients, with a protective effect of the minor allele (*G*) (OR 0.41, 95% CI 0.17–0.97, *p* = 0.025), which persisted after controlling for the Grace scale and age. This shows that the results of studies correlating new AF with genetic polymorphisms are inconsistent, probably because of the different populations studied. There are certainly not many large prospective and retrospective studies on new AF, and we think this may be an area we need to focus on in the future, and we look forward to more in-depth studies on this population.

## 4. Limitations and Outlooks

In the past decade, considerable progress has been made in identifying rare and common genetic variants associated with atrial fibrillation. However, there is still no conclusive evidence that the available data from genetic studies can be used for clinical decision-making in patients with atrial fibrillation. The reasons for this may be as follows: the first is the lack of a more comprehensive understanding of the potential genetic substrates of AF; the second is the fact that many genetic studies are based on retrospective studies and that extensive prospective randomized controlled studies are still lacking; and the third is the fact that there are differences by race and ethnicity and that many of the current study data are based on European, American, and Asian populations and populations in more economically developed regions. Although many retrospective studies have shown that outcomes after atrial fibrillation ablation are influenced by genetic variation, we consider the following points for improvement in how to apply the genetic findings to clinical practice better.

First, we found through extensively reading the literature that although different genetic variants or different variants of the same gene have some predictive value for outcome after AF ablation, it may be more reasonable to use the genetic risk score (GRS) as a risk assessment for patients with AF, more beneficial to identify patients at higher risk of developing AF, and thus more helpful to identify those prone to recurrence after AF ablation [146].

Second, many AF centers have data based on the more economically developed Caucasian and Asian populations, which is not representative of the general global population. In addition, we serve people from all levels of society and certainly from socioeconomically deprived areas. Hence, databases with broader applicable populations must be built, and more prospective randomized controlled studies must be conducted.

Third, the translation of genetic data to the clinic is also limited by technology. GWAS and GRS are only available in advanced vascular disease research centers. The ability to test quickly and obtain results is still debatable. Therefore, whether genetic testing can be made cheaper and more convenient through competitive mechanisms or technological improvements is a question that deserves to be addressed to enable its more comprehensive application.

## 5. Conclusions

Overall, although current research and cognitive gaps still need to be addressed, the application of genetics to the prediction of outcomes after AF ablation holds great promise. Both individual patients, their families, and even society as a whole will benefit from this.

## Search Strategy and Selection Criteria

This review used the Pubmed database to identify references for this review. The search terms covered all parts of this review, but were not limited to “atrial fibrillation recurrence” and “genetic polymorphisms”. Three authors (MFW, CX, and SYS) searched for the full text of these studies and performed an evaluation. Disagreements were resolved by discussion.
